# Strengthening the National Care System for Young Onset Dementia

**DOI:** 10.3389/ijph.2022.1605099

**Published:** 2022-11-03

**Authors:** Tomohiro Kogata

**Affiliations:** ^1^ Obu Center for Dementia Care Research and Practices, Obu, Japan; ^2^ Department of Integrated Health Sciences, Graduate School of Medicine, Nagoya, Japan

**Keywords:** dementia, care, public policy, young onset dementia, coordination

**The IJPH series “Young Researcher Editorial” is a training project of the Swiss School of Public Health.**

In our rapidly aging societies, developing a sustainable integrated care system for dementia is a critical health challenge. Dementia is a syndrome of cognitive deterioration caused by diseases or injuries (1, 2), and we tend to assume that people living with dementia are elderly, frail, or lack mobility. Young-onset dementia (YOD) (3) is less common, but not truly rare. A recent meta-analysis revealed that the worldwide age-standardized prevalence of YOD for people aged between 30 and 64 was about 119.0 per 100,000, which was considered to be higher than previously expected (4). Diagnosis of YOD is often delayed, preventing people living with YOD and their families from receiving appropriate care (5). They lose more lifetime income and have higher health costs because they must depend on chronic care for most of their lives. Therefore, we argue that a system of care should be formed to effectively support people living with YOD on their complex journey.

Some countries are focusing on care for YOD as well as dementia in the elderly (6, 7). Japan has designed policies and made practical guidelines for people living with YOD. There, a national project for people living with dementia has encouraged local governments to coordinate practices for YOD care. However, what type of care is best for people living with YOD and their families? Support systems may vary across countries, according to local situations and the needs of users.

In Japan, a YOD coordinator answers questions and acts as the hub for contact with people living with YOD, their families, caregivers, or health professionals. There are YOD coordinators in every prefecture (the area of local government) and some large cities. They provide support, information, and advice in the living community. The local government may commission appropriate staff in diverse facilities by selecting those capable of contributing to YOD care in the community. In each community, the YOD coordinators are responsible for: 1) providing information or organizing links between support personnel, 2) developing the collaborative support network in the community, and 3) planning activities to improve community care. YOD coordinators must connect the people living with YOD to services in the community. Given this background of the system, YOD coordinators consider the needs of people living with YOD and coordinate their access to services.

Similar care systems have been adopted in a few other countries, including Netherlands, which takes a hub-and-spoke approach (8), and Australia, which provides care through the country’s National Disability Insurance Scheme (NDIS) (9). Unlike other countries that do not have a YOD specialized care system, these countries have set up common regional service hubs at the local level. Netherlands has a long-established YOD care system in which national and regional centers play key roles in educating and training people living with YOD. The Dutch government recognized the need for YOD care and may further develop regional centers. Australia set up YOD key workers as service coordinators for community care, but abolished it in 2019, so now people living with YOD access care through the NDIS, which is available to people with severe and permanent disabilities at the local level (10).

Netherlands, Australia, and Japan have community hubs for YOD care in common, but they differ in whether they specialize in the disease of YOD. Japan and Netherlands have specialized service hubs for YOD, but Australia has inclusive service hubs accessible for all people with disabilities including people living with YOD. This difference may be the result of the flexibility of each country’s social welfare policy to address the care of YOD.

All countries must meet the common challenges of YOD care and provide needs-oriented and age-appropriate services. Currently, most services do not fit the needs of people living with YOD and their families due to the deferential needs of those who suffer in advanced age (11). Some people living with YOD need access to resources that help them reacquire meaningful roles and be re-employed for jobs. Tailored YOD services could provide one-stop support, and work in the aforementioned countries. To form an effective service system, YOD care should be integrated YOD specialized services into the existing government systems. Through the specialized care service, people living with YOD and their families may lead to meaningful engagement and increase their satisfaction with care.
